# Association between vitamin D and incident herpes zoster: a UK Biobank study

**DOI:** 10.3399/BJGP.2021.0623

**Published:** 2022-08-09

**Authors:** Liang-Yu Lin, Rohini Mathur, Amy Mulick, Liam Smeeth, Sinéad M Langan, Charlotte Warren-Gash

**Affiliations:** Faculty of Epidemiology and Population Health, London School of Hygiene and Tropical Medicine, London, UK.; Faculty of Epidemiology and Population Health, London School of Hygiene and Tropical Medicine, London, UK.; Faculty of Epidemiology and Population Health, London School of Hygiene and Tropical Medicine, London, UK.; Faculty of Epidemiology and Population Health, London School of Hygiene and Tropical Medicine, London, UK.; Faculty of Epidemiology and Population Health, London School of Hygiene and Tropical Medicine, London, UK.; Faculty of Epidemiology and Population Health, London School of Hygiene and Tropical Medicine, London, UK.

**Keywords:** electronic health records, herpes zoster, primary health care, UK Biobank, vitamin D

## Abstract

**Background:**

Vitamin D has immunomodulatory effects, but any association with herpes zoster (HZ) is unclear.

**Aim:**

To explore the association between vitamin D status and risk of incident HZ in adults in the UK.

**Design and setting:**

A cohort study involving participants of UK Biobank (a database containing the health information from half a million individuals) across England, Wales, and Scotland, who had at least one vitamin D testing result with linked primary care electronic health records.

**Method:**

The primary exposure was vitamin D status, categorised as deficient (<25 nmol/L), insufficient (25–49 nmol/L), or sufficient (≥50 nmol/L). The secondary exposures were self-reported vitamin D supplementation at baseline assessment and vitamin D prescription records. The outcome was diagnosed incident HZ, identified from linked primary care or hospital inpatient records. Weibull regression was used, adjusting for potential confounders, including demographic factors, comorbidities, and immunosuppression.

**Results:**

In total, 177 572 eligible participants were included in the analysis, with a mean follow-up time of 10.1 years (standard deviation 1.9 years). No evidence showed that low vitamin D was associated with a higher incidence of HZ, compared with people with sufficient vitamin D (deficient: adjusted hazard ratio [HR] 0.99, 95% confidence interval [CI] = 0.90 to 1.10; insufficient: HR 1.03, 95% CI = 0.96 to 1.10). No evidence was found that supplementing vitamin D or receiving vitamin D prescription was associated with HZ incidence (supplementation: HR 0.88, 95% CI = 0.67 to 1.16; prescription: HR 1.11, 95% CI = 0.91 to 1.34).

**Conclusion:**

No association of vitamin D status, supplementation, or prescription with incident HZ was observed. No evidence supported vitamin D supplementation as a strategy to prevent HZ.

## INTRODUCTION

Herpes zoster (HZ) is a common disease among adults. In the UK, its annual incidence is around five per 1000 person-years,^1^ and its average lifetime risk is around 30% in people without vaccination.^,^^2^ The typical symptoms are unilateral, painful, vesicular rashes in a dermatomal distribution that last for approximately 7–10 days. HZ greatly decreases patients’ quality of life, and substantially increases medical and social costs.^1^ It may also be associated with a range of neurological, ocular, cutaneous, and visceral complications.^3^ The most important risk factor for HZ is ageing, as immunity wanes over time.^4^ Immunosuppression and some comorbidities — such as chronic kidney disease (CKD) and systemic lupus erythematosus (SLE) — are also associated with increased HZ risk.^5^ Vaccines are effective at reducing the risk of HZ;^6^ however, in the UK, the vaccination programme is only available for people aged ≥70 years.^7^ Studying other possible preventive measures for HZ is important, especially for people aged <70 years.

The musculoskeletal protection effects of vitamin D, which regulates calcium and phosphate homeostasis, have been well established.^8^ In addition, the non-skeletal effects of vitamin D — such as immunomodulation — have been recently studied. In vitro studies have shown that vitamin D could stimulate the expression of antimicrobial peptides, protecting against infections;^9^^,^^10^ however, epidemiological studies have shown inconsistent associations between vitamin D and infections. A systematic review and meta-analysis published in 2021, combining 37 clinical trials of vitamin D supplementation, showed that taking vitamin D slightly decreased the risk of respiratory infections (odds ratio 0.92, 95% confidence interval [CI] = 0.86 to 0.99).^11^ The authors’ previous systematic review and meta-analysis found inconclusive evidence for any association between vitamin D and herpes viruses in studies conducted primarily among individuals who were immunosuppressed.^12^ One case–control study among people with CKD showed that vitamin D supplementation may decrease the odds of HZ.^13^

If vitamin D deficiency is associated with an increased risk of HZ in the general population, taking vitamin D supplements may become a cheap public-health measure for its prevention. As such, using the UK Biobank cohort, the authors aimed to explore the association between serum vitamin D status or supplementation and the risk of HZ.

**Table 1:** How this fits in

Vitamin D is regarded as having some antimicrobial effects. Large, nationwide cohort data were used to explore the association between vitamin D status and the risk of herpes zoster (HZ). The results showed that serum vitamin D status, vitamin D supplementation, and vitamin D prescriptions in primary care were not associated with incident HZ. Based on the evidence available, vitamin D supplements are not an effective intervention to prevent HZ.

## METHOD

### Data source and cohort

The data source was the UK Biobank database, which contains the records of approximately half a million participants, aged 40–69 years, from England, Wales, and Scotland, who were recruited between 2006 and 2010. At recruitment, these participants visited one of 22 UK Biobank assessment centres, in which they received physical examinations, completed questionnaires, and gave biological samples including of blood, urine, and saliva.^14^ Participants also consented to the linking of their clinical data, which included diagnosis codes for inpatient and outpatient visits, diagnosis dates, hospitalisation episode or consultation dates, and prescribing records from primary care.^15^^,^^16^ Nearly all participants (about 88%, 440 000 participants) have linked hospital inpatient records, and around 230 000 participants also have their primary care records linked.^17^

Participants were followed up from the date they visited the assessment centre, with the end of follow-up defined as the date when HZ was diagnosed, the date of death or loss to follow-up, or 31 July 2019 (whichever was first).

### Primary exposure: vitamin D status

The primary exposure of the study was serum vitamin D status recorded on recruitment to UK Biobank and when participants visited UK Biobank assessment centres between 2006 and 2010. The detailed methods for measuring serum vitamin D levels are described in Supplementary Box S1. In line with Public Health England’s classification,^18^ vitamin D levels were coded as follows:

- deficient (<25 nmol/L serum 25-hydroxyvitamin D);
- insufficient (25–49 nmol/L serum 25-hydroxyvitamin D); or
- sufficient (≥50 nmol/L serum 25-hydroxyvitamin D).

The demographic characteristics of the included participants were compared by their vitamin D status.

### Secondary exposure: vitamin D supplementation

A secondary exposure, vitamin D supplementation, was recorded using a self-reported questionnaire completed during each participant’s baseline visit to a UK Biobank assessment centre between 2006 and 2010. This included self-reported use of over-the-counter supplements, such as vitamin D, multivitamins, fish oil, and calcium.

An additional secondary exposure was GP-prescribed vitamin D supplementation, obtained with GP prescription data in the 2 years before the baseline assessment. The detailed data management of secondary exposures is summarised in Supplementary Box S2.

### Outcome

The outcome of the study was incident HZ, defined through a clinical diagnosis recorded in the linked primary care and inpatient records. Diagnosis code lists for HZ were developed using Read 2, Clinical Terms Version 3, and International Classification of Diseases (10th revision) (ICD-10) codes to identify incident HZ from the dataset.^15^^,^^16^ Incident HZ diagnoses were defined as participants with an HZ diagnosis occurring at least 1 day after the baseline assessment to 31 July 2019.

### Study eligibility

**Figure 1.**
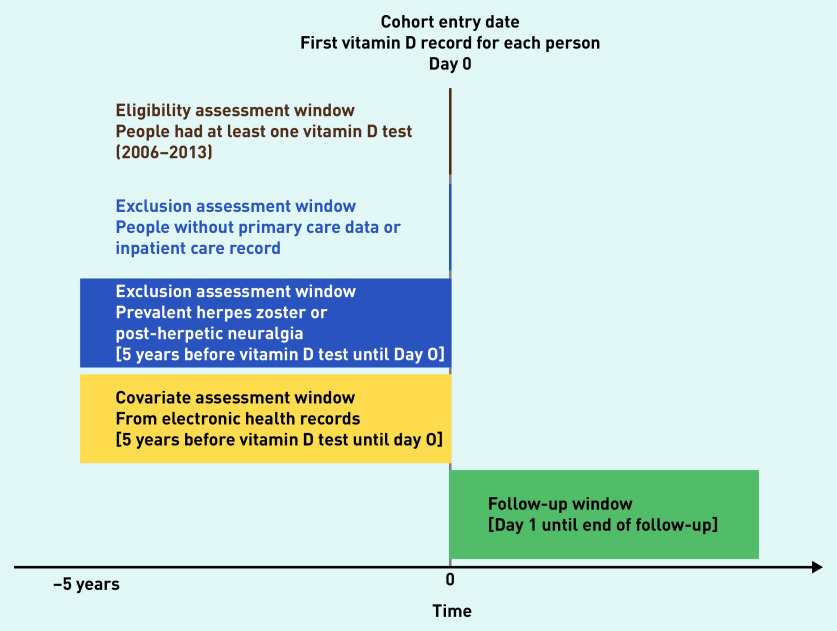
*Study design chart.*

The study design is summarised in Figure 1. UK Biobank participants were eligible if they had at least one vitamin D record, and both primary care and inpatient care records. Participants with no vitamin D record, with no linked electronic health record (EHR), or with previous HZ or post-herpetic neuralgia within 5 years before follow-up were excluded. Demographic data for included and excluded participants were compared.

### Measurement of covariates

Some demographic factors associated with vitamin D deficiency and insufficiency were recorded at baseline assessment, including sex, age, ethnicity, body mass index (BMI), smoking status, drinking frequency, Index of Multiple Deprivation (IMD) level, UK Biobank assessment centre region, and the seasons when vitamin D was tested.^19^ Comorbidities associated with an increased risk of HZ, such as CKD and SLE,^5^ were identified from the linked EHR and from self-reported health conditions. Severe immunosuppressive conditions — including organ transplantation, chemoradiotherapy, cell-mediated immunosuppression, HIV, blood cancers, chemotherapy (biological and non-biological agents), bone-marrow transplantation, and long-term oral steroid use — were identified solely from the clinical datasets. Long-term oral steroid use was defined as at least two issued steroid prescriptions within 90 days. These clinical covariates were defined as having had a diagnostic code in the 5 years before follow-up. For blood cancer, bone-marrow transplantation, and steroid use, the covariate assessment time windows were up to 2 years before follow-up.

### Statistical analysis

This was a historical cohort study. The association between the primary and secondary exposures and incident HZ were assessed using Weibull regression models, with adjustment for possible confounders that were selected by using a directed acyclic graph approach, summarised in Supplementary Figure S1. The models included sex, age, BMI, ethnicity, smoking status, drinking frequency, IMD scores, UK Biobank assessment centre region, vitamin D testing seasons, underlying comorbidities, and immunosuppression, which are described in Supplementary Box S3.

The number of participants with missing data was <3% across demographic and health-related factors; for self-reported vitamin D supplementation, however, 54.5% of data were missing. As the proportion of missingness in most demographic factors was low, and the chance of being a complete case was not dependent on the outcome, a complete-case analysis was performed that only included people without missing data in the models.^20^

All statistical analyses and plotting were performed using R statistical software (version 4.1.1).

### Sensitivity analysis

Various sensitivity analyses were performed; the justifications/rationales are summarised in Supplementary Table S1. Records dating from after September 2013, when the vaccination programme was introduced, were excluded, and the effects of different covariate definitions were compared. To eliminate the potential effect of time-varying hazards, analyses were rerun using the Cox proportional hazards regression model. Poisson regression was also used, assuming baseline hazards are constant.

## RESULTS

### Study population

**Figure 2.**
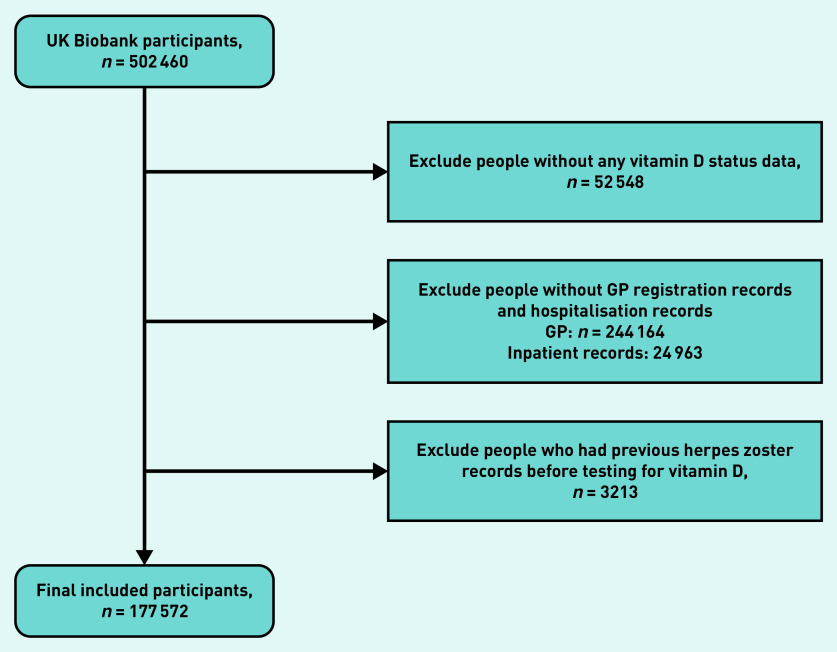
*Flowchart of sample selection.*

The study population selection process is outlined in Figure 2. A comparison of the included and excluded participants is summarised in Supplementary Table S2. The distribution of demographic factors was similar between the included and excluded participants, with the exception of assessment centre region: the proportions of people from Yorkshire and the Humber, Scotland, Wales, and East Midlands areas that were included in the analysis were far higher than those from other regions, while the proportions of those from the South East and South West were much lower.

After excluding people without vitamin D records, without clinical records, or with previous HZ episodes, 177 572 participants were included in the analysis. Of these, 6583 (3.7%) people died, 211 were lost to follow-up (0.1%), and 6616 (3.7%) had incident HZ diagnosis. Among the 6616 people with outcomes, more people had sufficient vitamin D levels (*n* = 2948, 45%) than those with insufficient (*n* = 2806, 42%) or deficient (*n* = 862, 13%) vitamin D. The proportion of missingness across demographic factors was <3%; 54.5% of self-reported vitamin D supplementations were missing (Supplementary Table S3).

The distribution of demographic factors by vitamin D status is summarised in Supplementary Table S4. Across different vitamin D statuses, the distributions of sex, age, comorbidities, and immunosuppression were similar. More participants with Asian or Black ethnic backgrounds were vitamin D deficient at baseline, compared with people with white ethnicity. Participants in the vitamin D deficient group were more likely to be obese, smoked more, and lived in more deprived areas compared with people with sufficient vitamin D; they also drank less frequently and were less likely to receive vitamin D prescriptions compared with people with sufficient vitamin D. More people with vitamin D deficiency were tested in winter compared with people with sufficient vitamin D, and more participants who were vitamin D deficient were from Scotland than from other countries and regions. The mean follow-up periods of people with different vitamin D statuses were similar (10 years).

### Association between vitamin D and risk of HZ

**Figure 3.**
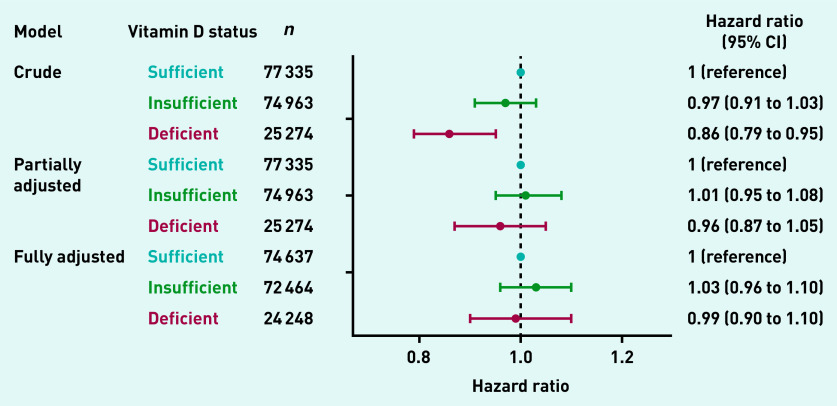
*Vitamin D status and the risk of incident herpes zoster. CI = confidence interval.*

The associations between vitamin D status and the risk of incident HZ are summarised in Figure 3. Compared with people with sufficient vitamin D status, some evidence existed that vitamin D deficiency was associated with a decreased risk of incident HZ in the crude Weibull regression model (hazard ratio [HR] 0.86, 95% confidence interval [CI] = 0.79 to 0.95). However, in models adjusted for sex and age, as well as in models fully adjusted for all covariates, no evidence showed that vitamin D deficiency or insufficiency were associated with incident HZ (partially adjusted model — insufficient vitamin D: HR 1.01, 95% CI = 0.95 to 1.08; deficient vitamin D: HR 0.96, 95% CI = 0.87 to 1.05; fully adjusted model — insufficient vitamin D: HR 1.03, 95% CI = 0.96 to 1.10; deficient vitamin D: HR 0.99, 95% CI = 0.90 to 1.10).

### Association between vitamin D supplementation and risk of incident HZ

**Figure 4.**
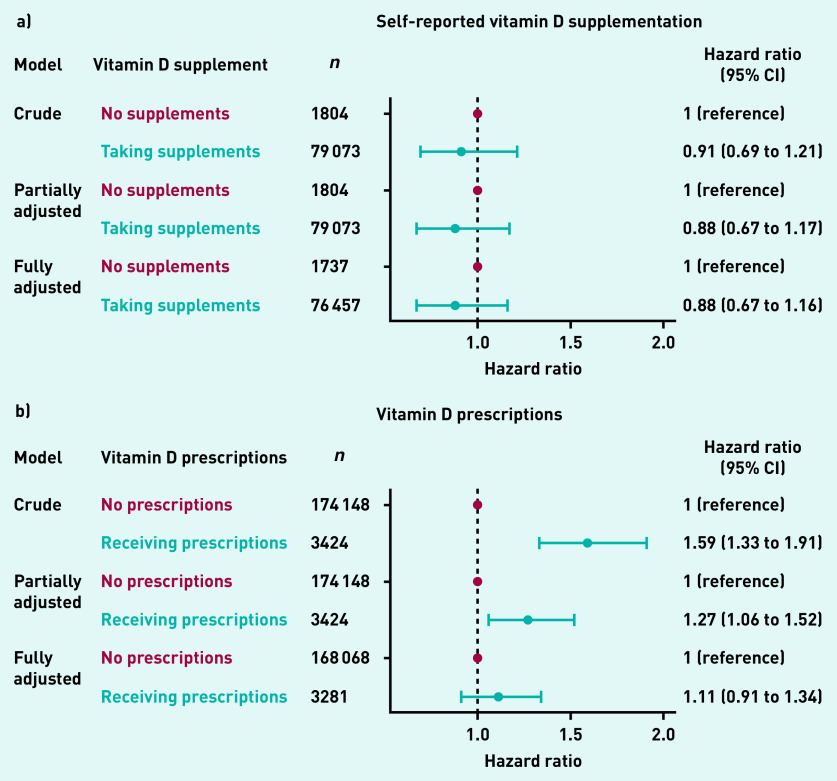
*Association between vitamin D supplementation and risk of incident herpes zoster for: a) self reported vitamin D supplementation; b) vitamin D prescriptions. CI = confidence interval.*

Figure 4a shows the association between self-reported vitamin D supplementation and the risk of incident HZ. The authors found no evidence that self-reported vitamin D supplement use was associated with incident HZ in the subgroup of participants for whom this information was recorded.

Some evidence existed that ever having received vitamin D prescriptions was associated with an increased risk of HZ in the crude model (HR 1.59, 95% CI = 1.33 to 1.91) and partially adjusted model (HR 1.27, 95% CI = 1.06 to 1.52); however, such association disappeared after fully adjusting for potential confounders (HR 1.11, 95% CI = 0.91 to 1.34) (Figure 4b).

### Sensitivity analyses

After excluding records dated after 1 September 2013, the main findings remained similar. Vitamin D deficiency or insufficiency (Supplementary Figure S2), vitamin D supplementation (Supplementary Figure S3a), or receiving vitamin D prescription (Supplementary Figure S3b) provided no evidence of an association with HZ. Different covariate definitions were compared, but the results did not differ from the initial model (Supplementary Figure S4). The stratified Cox regression model showed no evidence of an association between vitamin D status and incident HZ, either before or after the vaccination programme was initiated (Supplementary Figure S5). The Cox proportional-hazards model showed no evidence of an association between vitamin D supplementation and HZ (Supplementary Figure S6a); however, weak evidence existed that vitamin D prescription was associated with a higher risk of HZ after fully adjusting for potential confounding factors (adjusted HR 1.17, 95% CI = 1.00 to 1.37, Supplementary Figure S6b). The Poisson regression model showed no evidence of an association with vitamin D status (Supplementary Figure S7), or supplementation or prescription with HZ (Supplementary Figure S8).

## DISCUSSION

### Summary

No evidence of an association between vitamin D deficiency or insufficiency and incident HZ was found after adjusting for potential confounders. Self-reported vitamin D supplementation or receiving vitamin D prescriptions also showed no evidence of an association with incident HZ. The results were robust across a range of sensitivity analyses, such as excluding records during the vaccination period and adjusting for differing definitions of confounding factors.

### Strengths and limitations

The study has several strengths. Compared with previous, small, studies conducted with patients with clinically high risk of HZ, this large study of a general population provides greater statistical power and generalisability. In addition, the vitamin D levels were measured systematically, and the proportion of covariate missingness was relatively low. The linkage between UK Biobank and the primary and secondary care records also enabled participants to be followed up for a long time and incident cases to be identified.

Nevertheless, some limitations also need to be acknowledged. The exposure and some covariates are likely to be time dependent, but measures used were taken at baseline. In the authors’ previous analysis, the proportion of vitamin D deficiency was lowest in summer and more prevalent in winter and spring;^19^ in another study measuring vitamin D repeatedly, the intraclass correlation coefficient between two vitamin D measurements after 5 years was only 0.59, which was moderately reliable.^21^ In the analysis presented here, Weibull regression was used, which assumes hazards increase during follow-up, and adjustments were made for vitamin D testing seasons in the model to minimise the effect of seasonal variation. In the sensitivity analysis, Cox model regression was used to adjust for potentially time-varying hazards and the results remained similar. Nevertheless, vitamin D status would change over time, which may introduce exposure misclassification.

It should also be noted that, despite the completeness of most covariates, more than half of the data were missing for self-reported vitamin D supplementation; as such, this variable may not reflect the real vitamin D supplementation use, and its association with the outcome needs to be interpreted with caution. A positive trend of association between GP-prescribed vitamin D supplementation and HZ was noted in the crude and partially adjusted models in the sensitivity analysis. This association may be due to confounding by indication, as well as the underestimation of unreported food fortification. People receive vitamin D prescriptions to prevent or treat vitamin D deficiency, but that indication for the prescription was not considered in the study presented here. Vitamin D food fortification is another main source of vitamin D supplementation in the UK primary care setting;^22^ however, because of the limitation of data availability, it was not included in the analysis.

The outcomes may also be underascertained. HZ was defined using EHRs, but people with a greater number of comorbidities may visit their primary care physicians more frequently than people without underlying chronic diseases; as such, HZ in these people is more likely to be diagnosed, whereas mild shingles among a younger or healthier population might not be noticed.^23^ In the study population, although the proportions of people with certain conditions were slightly higher in those who were deficient in vitamin D, the overall distributions of comorbidities and immunosuppression were similar across different vitamin D statuses. Any ascertainment bias in the study presented here should be non-differential.

Misclassification of HZ outcomes cannot be ruled out. Studies using EHRs assume that individuals have a disease if they have the corresponding diagnostic codes; conversely, people without specific diagnostic codes are assumed not to have the disease. It is possible that some participants with HZ did not visit their GP or did not have a confirmed diagnosis, so their disease statuses might have gone unrecorded.

Regarding possible ascertainment bias, a study in the US reported that using the ICD-9 code for definite or possible herpes virus could identify 98% of HZ cases (sensitivity 98%), and the positive predictive value was also very high (PPV 93%).^24^ Furthermore, the financial barrier to access health care in the UK is generally lower than in the US, which may increase the sensitivity of HZ diagnoses in the EHR databases.

Residual confounding effects also cannot be ruled out. Using diagnostic codes from the linked records may underestimate the true prevalence of some diseases, such as CKD. In studies using EHRs, serum creatinine levels are more often used to diagnose CKD instead of using diagnostic codes alone;^25^ however, laboratory test results are not available in the linked EHRs of UK Biobank. To enhance the sensitivity of detecting comorbidities, the authors of the study presented here included self-reported, non-cancer health conditions in the analysis, but the overall prevalence of CKD was still much lower than the national prevalence during the same period.^26^

### Comparison with existing literature

This is, to the authors’ knowledge, the first published study assessing the association between vitamin D status and incident HZ in the general population. Previous studies on the topic have been conducted among people with immunosuppression; as an example, a case–control study about taking vitamin D supplements and HZ only included 126 patients with CKD, while the present study included more than 170 000 healthy participants.^13^ Compared with this previous study, the population of the study presented here was, largely, immunocompetent.

Another small, single hospital-based study in Taiwan compared people with post-herpetic neuralgia with matched hospital controls to assess the association between vitamin D and post-herpetic neuralgia. This cross-sectional analysis showed strong evidence that people with hypovitaminosis (defined as serum vitamin D <75 nmol/L) were associated with higher odds of having post-herpetic neuralgia (adjusted odds ratio = 3.12; 95% CI = 1.73 to 5.61).^27^ However, the cross-sectional analysis cannot distinguish temporality, which may lead to reverse causation. The authors’ cohort study has less potential for reverse causation, but it did not distinguish post-herpetic neuralgia from herpes zoster because of the limitation of the data.

### Implications for practice

This cohort study showed no evidence to support an association between vitamin D status or supplementation and incident HZ. Based on the evidence that is currently available, vitamin D testing, supplementation, or fortification cannot be recommended to prevent HZ.

## Data

Complete pseudonymised participant data are not openly shared under the material transfer agreement with UK Biobank and ethics approval. Other eligible researchers can apply for UK Biobank data to answer specific research questions. Relevant data information about the project can be accessed using the reference number ‘UK Biobank Application 51265’.
